# Supplementary material for: Exploring the association between social determinants and aphasia impairment: A retrospective data integration approach
Source: PLoS One. 2024 Mar 21;19(3):e0299979. doi: 10.1371/journal.pone.0299979 (PMC10956803; doi:10.1371/journal.pone.0299979)
Supplement: S1 Appendix — (DOCX) [file pone.0299979.s001.docx]

**APPENDIX**

| Table A1: Associations with Aphasia Impairment by Region | | | | | | | | |
| --- | --- | --- | --- | --- | --- | --- | --- | --- |
|  | Northeast | | | | Midwest | | | |
| R-Square | 0.8662 |  |  |  | 0.6497 |  |  |  |
| Adj R-Sq | 0.8242 |  |  |  | 0.6108 |  |  |  |
|  | Estimate | Std Error | t Value | Pr > \|t\| | Estimate | Std Error | t Value | Pr > \|t\| |
| Intercept | **4.572** | 0.171 | 26.680 | <.0001 | **4.420** | 0.078 | 56.770 | <.0001 |
| Age > 60 | -0.089 | 0.088 | -1.020 | 0.317 | -0.015 | 0.045 | -0.330 | 0.744 |
| Income > $30,000 | 0.114 | 0.072 | 1.590 | 0.122 | -0.044 | 0.048 | -0.920 | 0.362 |
| TPO | -0.013 | 0.042 | -0.310 | 0.755 | **0.054** | 0.027 | 2.010 | 0.047 |
| Family Size | -0.021 | 0.038 | -0.550 | 0.586 | -0.027 | 0.020 | -1.360 | 0.177 |
| Female | -0.022 | 0.079 | -0.280 | 0.784 | 0.050 | 0.045 | 1.120 | 0.266 |
| Black | 0.045 | 0.105 | 0.430 | 0.669 | **-0.013** | 0.056 | -2.240 | 0.018 |
| Hispanic | 0.231 | 0.183 | 1.260 | 0.216 | -0.032 | 0.125 | -0.260 | 0.796 |
| Uninsured | 0.186 | 0.212 | 0.880 | 0.386 | -0.025 | 0.073 | -0.340 | 0.735 |
| College Degree | -0.003 | 0.106 | -0.030 | 0.977 | 0.033 | 0.100 | 0.330 | 0.741 |
| Global Aphasia | **-1.379** | 0.115 | -11.980 | <.0001 |  |  |  |  |
| Broca's Aphasia | **-0.591** | 0.072 | -8.170 | <.0001 | **-0.488** | 0.042 | -11.730 | <.0001 |
|  | South | | | | West | | | |
| R-Square | 0.7523 |  |  |  | 0.6689 |  |  |  |
| Adj R-Sq | 0.7325 |  |  |  | 0.6203 |  |  |  |
| F-Value | 38.09 |  |  |  | 13.77 |  |  |  |
|  | Estimate | Std Error | t Value | Pr > \|t\| | Estimate | Std Error | t Value | Pr > \|t\| |
| Intercept | **4.351** | 0.042 | 103.430 | <.0001 | **4.319** | 0.068 | 63.330 | <.0001 |
| Age > 60 | -0.030 | 0.028 | -1.060 | 0.290 | 0.021 | 0.051 | 0.410 | 0.681 |
| Income > $30,000 | 0.017 | 0.030 | 0.550 | 0.583 | 0.014 | 0.056 | 0.260 | 0.798 |
| TPO | **0.049** | 0.017 | 2.920 | 0.004 | **0.071** | 0.030 | 2.400 | 0.019 |
| Family Size | 0.010 | 0.010 | 1.050 | 0.294 | 0.013 | 0.016 | 0.800 | 0.427 |
| Female | 0.006 | 0.026 | 0.240 | 0.813 | 0.026 | 0.050 | 0.520 | 0.604 |
| Black | **-0.064** | 0.046 | -0.018 | 0.046 | 0.108 | 0.088 | 1.230 | 0.221 |
| Hispanic | -0.013 | 0.078 | -0.170 | 0.869 | **-0.025** | 0.132 | -0.019 | 0.049 |
| Uninsured | 0.018 | 0.073 | 0.250 | 0.806 | -0.138 | 0.177 | -0.780 | 0.440 |
| College Degree | 0.069 | 0.045 | 1.530 | 0.129 | -0.034 | 0.132 | -0.260 | 0.796 |
| Global Aphasia | **-1.480** | 0.101 | -14.700 | <.0001 | **-1.457** | 0.346 | -4.210 | <.0001 |
| Broca's Aphasia | **-0.433** | 0.028 | -15.550 | <.0001 | **-0.536** | 0.049 | -10.850 | <.0001 |
| Dependent Variable: WAB-AQ | | | | | | | | |
| Estimates weighted to reflect nationally representative population and adjust for matched sample | | | | | | | | |
| ***Indicates*** significant at 95% confidence level. | | | | | | | | |
| Reference Category: Age (≤60), Income (low income ≤$30,000+), Insurance (insured), Race (White), Ethnicity (Non-Hispanic), Sex (Male), Aphasia Type (Anomic), Education (Less than a college degree) | | | | | | | | |

| Table A2: Associations with Aphasia Impairment by Age | | | | | | | | |
| --- | --- | --- | --- | --- | --- | --- | --- | --- |
|  | Age ≤ 60 | | | | Age > 60 | | | |
| R-Square | 0.6636 |  |  |  | 0.806 |  |  |  |
| Adj R-Sq | 0.6366 |  |  |  | 0.7931 |  |  |  |
|  | Estimate | Std Error | t Value | Pr > \|t\| | Estimate | Std Error | t Value | Pr > \|t\| |
| Intercept | **4.259** | 0.069 | 61.860 | <.0001 | **4.333** | 0.042 | 104.110 | <.0001 |
| Income > $30,000 | **0.006** | 0.035 | 0.017 | 0.039 | **0.024** | 0.027 | 0.019 | 0.037 |
| TPO | **0.076** | 0.020 | 3.800 | 0.000 | **0.058** | 0.016 | 3.670 | 0.000 |
| Family Size | 0.014 | 0.013 | 1.070 | 0.286 | 0.002 | 0.009 | 0.220 | 0.830 |
| Female | -0.021 | 0.031 | -0.990 | 0.322 | **0.092** | 0.026 | 3.560 | 0.001 |
| Black | **-0.031** | 0.038 | -0.018 | 0.042 | 0.026 | 0.061 | 0.430 | 0.670 |
| Hispanic | 0.050 | 0.112 | 0.450 | 0.654 | 0.031 | 0.062 | 0.490 | 0.623 |
| Uninsured | -0.100 | 0.074 | -1.350 | 0.177 | 0.018 | 0.056 | 0.310 | 0.755 |
| College Degree | 0.037 | 0.050 | 0.740 | 0.463 | 0.008 | 0.052 | 0.150 | 0.877 |
| Global Aphasia | **-1.000** | 0.124 | -8.070 | <.0001 | **-1.423** | 0.068 | -21.050 | <.0001 |
| Broca's Aphasia | **-0.430** | 0.031 | -13.990 | <.0001 | **-0.514** | 0.026 | -19.410 | <.0001 |
| Midwest | 0.044 | 0.050 | 0.890 | 0.375 | -0.033 | 0.041 | -0.810 | 0.417 |
| South | 0.071 | 0.048 | 1.500 | 0.135 | -0.005 | 0.036 | -0.150 | 0.878 |
| West | 0.024 | 0.054 | 0.440 | 0.658 | -0.001 | 0.040 | -0.010 | 0.989 |
| Dependent Variable: WAB-AQ | | | | | | | | |
| Estimates weighted to reflect nationally representative population and adjust for matched sample | | | | | | | | |
| ***Indicates*** significant at 95% confidence level. | | | | | | | | |
| Reference Category: Income (low income ≤$30,000+), Insurance (insured), Race (White), Ethnicity (Non-Hispanic), Sex (Male), Aphasia Type (Anomic), Education (Less than a college degree), Region (Northeast) | | | | | | | | |

| Table A3: Associations with Aphasia Impairment by Income | | | | | | | | |
| --- | --- | --- | --- | --- | --- | --- | --- | --- |
|  | Income < $30,000 | | | | Income ≥ $30,000 | | | |
| R-Square | 0.7308 |  |  |  | 0.7899 |  |  |  |
| Adj R-Sq | 0.7182 |  |  |  | 0.7554 |  |  |  |
|  | Estimate | Std Error | t Value | Pr > \|t\| | Estimate | Std Error | t Value | Pr > \|t\| |
| Intercept | **4.309** | 0.047 | 92.010 | <.0001 | **4.516** | 0.068 | 66.250 | <.0001 |
| Age > 60 | -0.027 | 0.025 | -1.090 | 0.277 | -0.019 | 0.036 | -0.550 | 0.587 |
| TPO | **0.049** | 0.014 | 3.430 | 0.001 | 0.035 | 0.021 | 1.640 | 0.104 |
| Family Size | 0.016 | 0.008 | 1.940 | 0.054 | **-0.047** | 0.018 | -2.540 | 0.013 |
| Female | 0.043 | 0.023 | 1.900 | 0.058 | 0.015 | 0.036 | 0.410 | 0.681 |
| Black | **-0.025** | 0.033 | -2.740 | 0.046 | 0.132 | 0.077 | 1.710 | 0.091 |
| Hispanic | **-0.008** | 0.060 | -2.140 | 0.049 | 0.084 | 0.158 | 0.530 | 0.596 |
| Uninsured | -0.043 | 0.048 | -0.880 | 0.377 | -0.330 | 0.169 | -1.950 | 0.054 |
| College Degree | 0.071 | 0.064 | 1.100 | 0.274 | 0.037 | 0.044 | 0.850 | 0.397 |
| Global Aphasia | **-1.306** | 0.071 | -18.500 | <.0001 | **-1.349** | 0.120 | -11.240 | <.0001 |
| Broca's Aphasia | **-0.481** | 0.023 | -20.890 | <.0001 | **-0.482** | 0.037 | -13.030 | <.0001 |
| Midwest | 0.033 | 0.039 | 0.850 | 0.398 | -0.081 | 0.057 | -1.430 | 0.156 |
| South | 0.039 | 0.035 | 1.110 | 0.267 | 0.001 | 0.050 | 0.030 | 0.977 |
| West | 0.024 | 0.038 | 0.640 | 0.524 | 0.021 | 0.064 | 0.320 | 0.747 |
| Dependent Variable: WAB-AQ | | | | | | | | |
| Estimates weighted to reflect nationally representative population and adjust for matched sample | | | | | | | | |
| ***Indicates*** significant at 95% confidence level. | | | | | | | | |
| Reference Category: Age (≤60), Insurance (insured), Race (White), Ethnicity (Non-Hispanic), Sex (Male), Aphasia Type (Anomic), Education (Less than a college degree), Region (Northeast) | | | | | | | | |
