# Supplementary material for: Exploring the association between social determinants and aphasia impairment: A retrospective data integration approach
Source: PLoS One. 2024 Mar 21;19(3):e0299979. doi: 10.1371/journal.pone.0299979 (PMC10956803; doi:10.1371/journal.pone.0299979)
Supplement: Attachment — Submitted filename: RESPONSE WITH REVIEWERS COMMENTS2.docx [file pone.0299979.s002.docx]

**General Editorial Comments:**

Please ensure that your manuscript meets PLOS ONE's style requirements, including those for file naming.

**We have reviewed and ensure the manuscript meet’s the journal style requirements.**

Please review your reference list to ensure that it is complete and correct. If you have cited papers that have been retracted, please include the rationale for doing so in the manuscript text, or remove these references and replace them with relevant current references.

**The reference list has been reviewed as recommended.**

The PLOS Data policy requires authors to make all data underlying the findings described in their manuscript fully available without restriction, with rare exception (please refer to the Data Availability Statement in the manuscript PDF file). The data should be provided as part of the manuscript or its supporting information, or deposited to a public repository.

**We have been responsive to the data policy as outlined in the appropriate section.**

Response to Reviewers

Reviewer #1: I only have minor comments. Line 97: has enabled researchers to researchers -> has enabled researchers.

**Revised as recommended.**

For table 2, it would be more reader-friendly if it adheres to the standard baseline table format.

**The table has been revised as recommended.**

Reviewer #2:

My only minor comment is for the authors to report effect sizes and the SE’s in the same format, e.g. 2.5% change (SE = 3.3%).

**This change has been made as recommended.**
